# Changes in Children’s Speech and Language Difficulties from Age Five to Nine: An Irish National, Longitudinal Study

**DOI:** 10.3390/ijerph18168483

**Published:** 2021-08-11

**Authors:** Roy McConkey, Ann Swift, Jill Titterington

**Affiliations:** 1 Institute of Nursing and Health Research, Ulster University, Newtownabbey BT37 0QB, UK; j.titterington@ulster.ac.uk; 2 School of Social Work and Social Policy, Trinity College Dublin, Dublin 2, Ireland; aswift@tcd.ie

**Keywords:** speech difficulties, language difficulties, children, longitudinal, national, survey, Ireland, speech and language therapy

## Abstract

In many countries, information on the prevalence of persistent speech and language disorders in early childhood is sparse due to the lack of nationally representative samples and longitudinal studies. Secondary analysis of data collected on over 7500 Irish children at ages 5 and 9 years, found that the prevalence of speech and language difficulties reported by the primary caregivers of Irish children decreased from one in six at age 5 to one in 12 at age 9. However, one in 20 children were reported to have difficulties at both ages. Regression analysis compared children with difficulties at both age 5 and age 9 to those who had been reported to have them at age 5 but no longer had such difficulties at age 9. Children with speech and language difficulties at both age 5 and age 9 were more likely to have two or more developmental impairments as well as current or past hearing impairments. Teachers and parents also reported a greater number of social-emotional difficulties. Family characteristics did not differ significantly across the two groupings. At best, up to one third of the children at ages 5 and 9 with speech and language difficulties had two or more contacts with a speech and language therapists in the preceding 12 month period. Increased support to these children, their parents and teachers would seem to be warranted.

## 1. Introduction

Speech and language difficulties in early childhood can have lasting impacts on a child’s educational achievements and social relationships [1] which may also extend into adulthood as well as affecting their occupational status [2]. However recent information on the population prevalence of speech and language disorders in children is limited as past studies have tended to focus on clinical or convenience samples at specific ages with few longitudinal studies, although there are notable exceptions [3,4,5]. Raghavan et al. [6] concluded from their review that “existing sources of data are inadequate to establish reliable rates of incidence, prevalence, and outcomes for speech and language disorders at the population level” (p. 1).

A further issue has been inconsistency in how speech and language disorders are assessed within population studies. The absence of short, robust screening tools [7] has forced researchers to rely largely on parental reports. However comparative studies between parental reports and professional assessment have shown sizeable correspondence [8,9]. Studies over the past 20 years and from various countries reported rates of speech and language difficulties of around one in ten among children aged three to seven years [10,11,12]. Moreover, a more sensitive indicator is the proportion of children for whom parents report persistent speech and language problems as their children age. A Norwegian study that examined changes from three to five years, identified that this was present in 3% of children, with 5% no longer having problems at five years of age but a further 6.5% reported to have problems at age 5 but not at age 3 [5]. Furthermore, the developmental trajectory of children with speech and language difficulties from five to eight years suggests a continuing gap with typically developing peers [13]. Contrasting the characteristics of children who seemed to improve with those with persistent problems would further identify those at greatest risk of having more severe speech and language difficulties and an increased need for additional support from teachers and therapists [3,14]. 

Past research suggests that boys are more likely to have speech and language difficulties. Additionally, a co-morbidity with other developmental impairments, such as intellectual disability and hearing loss, is well established [15,16]. In addition, associations between children’s behaviour problems and social-emotional difficulties have been identified [17,18] along with increased likelihood of bullying and poor peer relationship [13,19]. Equally family circumstances also appear to have an influence such as lower levels of socio-economic status and parental level of education, and poorer interactions with the child [3,6,20]. Thus, speech and language difficulties must be considered in the context of the child’s holistic development. However, the relative contribution of all these factors to children’s speech and language difficulties has rarely been examined, in part due to the large samples of children needed to undertake the required statistical analyses. 

An ongoing, national longitudinal study in the Republic of Ireland involving a birth cohort of over 11,000 children who were followed up at five and nine years of age, provided an opportunity to address some of the foregoing shortcomings in past research. It also provided the opportunity to identify the contacts which the children had with speech and language therapists in the preceding 12 months. Other studies have noted difficulties in accessing these therapies and the unmet training needs of teachers and therapists [4,21].

### Aims of Study

1. To describe the speech and language difficulties parents report when their children are five years of age and again when they are nine years of age.
2. To identify the changes in these difficulties over a five-year period.
3. To investigate the variables associated with improvements and continuing difficulties experienced by children at 9 years of age.
4. To identify the number of contacts that the child had with speech and language therapists.

The study is based on secondary analysis of anonymised children and family data that were obtained as part of a longitudinal study: called Growing Up In Ireland. Full details of the study and the measures used are available at: https://www.growingup.ie/. The data is publicly available through the Irish Social Sciences Database: https://www.ucd.ie/issda/.

## 2. Materials and Methods

### 2.1. Country Context

Ireland had a population of 4.59 million with 66,700 children aged 5 in 2013 and 71,300 aged nine in 2018. Children generally commence free primary education before age 5. Pupils with additional needs can be referred by parents and schools for assessment and therapeutic services provided by community health services. Ireland is classed by the World Bank as a high-income country. 

### 2.2. Growing Up in Ireland Longitudinal Study

The longitudinal study commenced in 2008/09 when the children were nine months of age and subsequently followed up when they were three, five and nine years of age. At nine months of age, a total of 11,134 children were recruited representing a response rate of 65% of all families approached and 69% of valid contacts were made in the course of the fieldwork. The families were chosen randomly from the list of the 41,185 infants registered on the ‘Child Benefit Register’—a universal benefit payable to each child in an Irish family—born between 1 December 2007 and 30 June 2008 [22]. 

The obtained sample was weighted at each data collection point to ensure that the structure of the completed sample at each age point was in line with the original population from which it has been selected. A total of 11 main characteristics of the infant and his/her family were used in the generation of the weights. As is common with longitudinal studies, sample attrition occurred so that by age 5 there were 9793 children (81% of the original sample) and at 9 years there were 8302 participants (72% of the starting sample). For the purposes of this study, information was available for 7700 children and families whose data was complete at ages 5 and 9 years. 

### 2.3. Information Gathered 

The initial contact with the family was made via a letter and information sheet from the Study Team. The interviewer subsequently made a personal visit to each household to arrange a face-to-face interview. At that first visit, interviewers asked to speak to the study child’s primary carer (usually the mother but fathers and grandmothers also had this role). After answering any queries the informant had, the interviewer asked the primary carer to sign two copies of the main consent form; one of which the carers retained. Only after securing signed consent did the interviewer commence the information gathering. This included an interview with the child’s primary carers (using computer assisted personal interviewing via a lap-top) and self-completion questionnaires with the child’s primary carer (using computer assisted self-completion software on a lap-top). In addition, self-completion questionnaires were sent, with parental permission, to the child’s teacher to obtain further information about the child. These were completed in writing and posted back to the Study Team. 

The questions were developed in consultation with various international experts and local personnel, with revisions undertaken in the light of their use in earlier waves of data gathering. Pilot testing was also undertaken of the measures prior to their use with each age group. The remainder of this section describes the information obtained at ages 5 and 9 that was pertinent to this study.

### 2.4. Information Pertinent to the Research Aims

Demographic information about the family obtained when the child was 5 years old was updated at age 9 and included details of lone parenting, the educational level of the primary carer, the socio-economic background of the family and the equivalized income level. The language used at home was not recorded but for nearly all families, this was likely to be English. 

In their interview, primary carers were presented with a list of possible speech and language difficulties commonly reported in children and asked to indicate if each applied to their child (see Table 1 given later). Primary carers could add additional difficulties their child experienced. A count was made of the number of different difficulties parents reported (range 0 to 10) so that changes from 5 to 9 years could be assessed statistically. Three grouping were identified: no speech and language difficulties; one or two reported difficulties and three or more reported difficulties (range 3 to 10). 

Primary carers were also asked at ages 5 and 9 if their child currently had, or at any time in the past, had any sort of hearing problem requiring correction. Children were then grouped into those who currently had a problem, those who had a problem in the past and those who had no problem. 

In addition, details were sought from primary carers of how many times, in the past 12 months, the child had seen a Speech and Language Therapist (SLT) or the primary carer had telephone contact. Based on the responses, three groupings were created: no contact with SLT; one contact with SLT (likely to be an assessment session) and two or more contacts with SLT (range 2 to 10+ contacts) in which face-to-face therapies are likely to have been undertaken. 

The following measures were also completed. 

#### 2.4.1. Children’s Developmental Impairments

Teachers were asked to indicate from a list of developmental impairments those that “limit the kind or amount of activity the study child can do at school”. The list included (with numbers and percentages added for the sample at nine years of age): Physical disability or visual or hearing impairment (*n* = 199:3.0%); Autism spectrum disorders (*n* = 223:3.4%); General learning disability (mild) (*n* = 339:4.5%); General learning disability (moderate/severe/profound) (*n* = 114:1.7%); Specific learning difficulties (e.g., dyslexia) (440:6.7%); and Emotional or behavioral problem (e.g., Attention Deficit (Hyperactivity) Disorder—ADD, ADHD) (*n* = 273:4.2%). Three groupings were created based on the frequencies of children reported as having each problem: children with no developmental impairments; those with one impairment reported and those with two or more impairments reported (range 2 to 6) as an indicator of co-morbidities. 

#### 2.4.2. Strengths & Difficulties Questionnaire (SDQ) [23]

The SDQ is a widely used, brief (25 item) behavioral screening questionnaire designed to assess emotional health and problem behaviours in children. A total score is calculated based on four of the five subscales with a cut-off score of 14 and above indicative of ‘possible problems’ and scores of 17 and over and requiring further investigation. The scale was completed by the primary carer when the child was aged 5 and again at 9, and also by the child’s teacher when the child was aged 9. On each assessment, the children were grouped into those above and below the two cut-off scores. 

The Cronbach alpha for the total scale score in this study was 0.74 for parents and 0.87 with teachers [17]. 

#### 2.4.3. The Pianta Scales—Child Parent Relationship Scale (CPRS) and Student-Teacher Relationship Scale (STRS) [24]

Each of these 15-item scales assesses the positive and negative aspects of the relationship between either the primary carer and child or teacher and child by using the same items for each. Two summary scores are derived: closeness and conflicting relationships. The scales were completed by the primary carer at age 5 and again at age 9 and by the child’s teacher at age 9. The Cronbach alpha for the primary carers were: Closeness 0.67: Conflict 0.79. The alpha for teachers were: Closeness 0.83: Conflict 0.86. 

#### 2.4.4. Parental Stress Scale [25]

A six-item Parental Stressors sub-scale from the above test was used with primary carers using a five-point rating. A total score was calculated with high scores indicative of greater stress. The Cronbach alpha for this subscale was 0.78.

#### 2.4.5. Centre for Epidemiological Studies Depression Scale (CES-D) [26]

These eight questions provide a short, self-report screening instrument for depression in the general population. Primary carers answered these items as part of the self-complete questionnaires. Higher scores are indicative of depression. The Cronbach alpha was 0.85 in this sample. 

### 2.5. Data Analysis

The information obtained when the children were aged 5 was merged with that obtained at age 9 on an SPSS database. Weightings were applied in accord with the algorithm developed for the Growing Up in Ireland study. This means that the sample sizes reported throughout this paper are slightly different from the information gathered at each age level. 

Using SPSS (vers 25), frequency counts and bivariate analyses were undertaken including Chi Sq tests and T-tests as appropriate. Effect sizes were also estimated. Binary Logistic Regression was used to identify the predictors of children for whom speech and language difficulties were reported at age 5 but who had none reported at age 9 (improvers) and those who had continuing difficulties. 

## 3. Results

### 3.1. Children’s Speech and Language Difficulties 

Table 1 presents the number and percentages of children reported by primary carers to have each of the difficulties listed at ages 5 and 9 (Note: These have been re-ordered in terms of the most common difficulties reported at age 5 and are based on a matched and weighted sample of 7689 cases). The small number of children reported to have other difficulties suggests that the listing captured the range of speech and language problems of which the primary carers were aware. 

As Table 1 shows, speech difficulties were the most common at both ages but by age 9, all these difficulties had reduced markedly. The decrease was more marked for speech difficulties and less so for language, such as finding words or putting words together. At age 5, 83.3% of children (*n* = 6409) had no speech and language difficulties reported by their primary carers. However, 5.7% (*n* = 439) were reported to have one difficulty and 10.9% (*n* = 841) had two or more difficulties. By age 9 for the same children, 92.0% had no reported difficulties, 2.9% had one difficulty and 5.2% had two or more difficulties. 

Boys outnumbered girls with speech and language difficulties: at age 5, 21.1% of boys had one or more difficulties reported compared to 12.0% of girls (Chi Sq = 127.7: *p* < 0.001) while at age 9, the comparable percentages were 10% boys and 5.6% girls (Chi Sq = 57.04: *p* < 0.001). 

Table 2 summarizes the changes in speech and language difficulties that had occurred between ages 5 and 9. In all, four out of five children had no difficulties reported at ages 5 and 9 but conversely one in five had at least one difficulty at these ages. 

As the table further shows, 895 children (11.7% of total sample) had no reported difficulties at age 9, although one or more had been reported by primary carers when the children were aged 5. Hence, these children could be considered to have improved. 

By contrast a total of 617 children had difficulties recorded at age 9. These included 250 children who were reported to have the same number of difficulties at both ages; 95 children who had reduced from 3+ difficulties to 1–2 difficulties and conversely 39 children who had increased from 1–2 difficulties to 3+ difficulties. Finally, 233 nine-year-olds were reported to have difficulties which had not been reported at age 5. 

### 3.2. Predictors of Change

In order to explore possible predictors of change, a comparison was made between children who had improved from age 5 and those who had continuing difficulties at 9 years of age. 

Various bi-variate analyses were undertaken to identify the variables that were significantly associated (*p* < 0.01) with children who had improved (*n* = 882:11.5% of the total sample) and those with difficulties at age 9 (*n* = 588:7.7% of the total. The numbers are reduced slightly due to missing data on certain predictor variables). Table 3 summarises the results of the statistical tests which were significant along with the effect size and the relevant summary scores for the variables tested, taken at age 9 unless otherwise stated. 

In addition, the variables that were not significantly associated with changes were the child’s gender, lone parenting, the primary carers’ educational level, socio-economic background of the family and equivalized household income (further details are available from the authors).

In order to control for the inter-relationships among these variables, a Binary Logistic Regression was used to assess the contribution of each variable to differentiating children who had improved from those who had continuing difficulties. Table 4 summarises the variables that contributed to the final regression model by each of the three model iterations. The variables were entered in discrete blocks to identify the contribution that each set of variables contributed to the regression model. Block one consisted of information gathered when children were aged 9. Block two added in primary carers’ report of the child being bullied and the number of friends also at age 9 as past research had identified these as being associated with children’s speech and language difficulties [13,19]. Block three were the variables relating to teacher ratings at age 9. The latter would identify if certain relationships held in school as well as home settings. A further block of information that had been obtained when the child was 5 years old was not included in the final analysis as it did not add significantly to the final model. Table 4 gives the model statistics as each block is added along with the amount of variance accounted for by the addition of further blocks (Nagelkerke R Square).

The regression analysis identified three significant indictors of children (*p* < 0.01) who had continuing speech and language difficulties at age 9 (see also Table 3). First, children with developmental impairments were more likely to have speech and language problems; with increased odds ratios of 1.6 with one impairment and 2.9 with two or more impairments. Second, children with past or current hearing problems were over twice as likely to have speech and language difficulties at age 9. Third, children with above threshold scores on social and emotional difficulties as rated by teachers also had higher odds ratios, but the ratings given by primary carers no longer contributed significantly to the final regression model.

In addition, the stress scores of primary carers also contributed to the model but to a lesser extent. Further, children who were reported to have been bullied did have slightly increased odds of having speech and language difficulties at age 9.

As noted above, none of the measures reflective of interactions between primary carers and children at age 5 contributed significantly to the regression.

However, the model, although statistically significant, only accounted for a small amount of the total variance (15%), which suggests a sizeable amount of the variation among children with speech and language difficulties at age 9 remains unexplained.

### 3.3. Contact with Speech and Language Therapists

In Ireland, as in many other countries, many children with speech and language difficulties are usually referred to a speech and language therapist (SLT) for assessment and therapy. Table 5 summarises the percentage of children when aged 5 and again at 9 who had one or more contacts with a SLT in the past 12 months for the groups of improvers and children with continuing difficulties.

Around half of the children at age 5 in both the improved and continuing difficulties groups had not seen a SLT in the preceding 12 months. A slightly higher proportion of improvers had one contact with an SLT whereas those with continuing difficulties had two or more contacts (92 of whom had 10 or more contacts). By contrast at age 9, those children with continuing difficulties were likely to have had either one contact or even more likely to have two or more contacts (54 of the 159 had 10 or more contacts). As the findings from the regression analysis in Table 4 imply, 66% of the 113 children who had one contact with an SLT at age 9 and 86% of the 159 children who had two or more contacts had developmental impairments and/or current hearing problems. Nevertheless, over half of the children with speech and language difficulties at age 9 had no contact with an SLT in the past 12 months.

## 4. Discussion

Few studies have examined changes in children’s speech and language difficulties. The analyses reported here describe the changes reported by parents when their children were aged 5 and again when they were nine years of age. The variables associated with improvements and continuing difficulties experienced by children at 9 years of age were also identified.

The main strength of the present study is the information gained on a nationally representative sample of over 7000 Irish children at two ages. An analysis could be made of children whose speech and language difficulties had abated between these ages and those who continued to experience difficulties. At five years of age around one in six children were reported by primary carers to have a speech or language difficulty, which is in line with prevalence rates reported in other countries [5,9,10]. However, by nine years, this number had dropped to 1 in 12 children; mostly accounted for by fewer speech difficulties reported rather than language delays as noted in Table 1. A further wave of data gathering in Ireland is planned for when the children are 13 years, which would clarify if the prevalence of speech and language difficulties continues to fall.

The regression analyses identified the co-morbidities that were associated with speech and language difficulties of children reported by parents at age 9. In particular, children with two or more developmental impairments were nearly three times more likely to have problems at age 9 compared to those children who no longer had problems. Likewise, children with hearing difficulties were also over twice as likely to have problems at age 9 as were those children whom teachers in particular had rated as having social and emotional difficulties. Each of these co-morbidities contributed to the likelihood of nine-old year children having speech and language problems. Thus, children with a combination of these three co-morbidities are likely to have a much greater risk of experiencing speech and language difficulties. Although past research had identified these co-morbidities as risk factors, this study confirms their cumulative contribution to children’s speech and language difficulties.

Contrary to some past research, though [14], in our analyses boys were no more likely than girls to have speech and language difficulties at age 9 compared to those who previously were reported to have them at age 5. Similarly, family factors such as primary carers’ educational level, socio-economic background of the family and lone parenting had no statistically significant effect. It may be that some of these family characteristics are more associated with the significant co-morbidities identified in the regression model. If past studies have not controlled for these co-morbidities in their analyses, they could wrongly associate them with speech and language difficulties.

Higher stress scores of primary carers did carry a small increased risk of children’s speech and language difficulties at age 9, as did reports of bullying but not higher depression scores of primary carers or ratings of conflicted relationship with the child. Additionally, the measures of parental wellbeing and conflicted interactions with the children when they were aged 5 had no significant association with later speech and language difficulties.

Nonetheless, the present findings should be interpreted cautiously as the amount of variance accounted for by the regression model is small and the increased risks identified are relatively modest. This suggests that other variables may influence children’s speech and language difficulties at age 9 and more especially that certain ones–including those considered in this study–could be associated with particular speech and/or language difficulties. Past studies suggest that there is a high degree of individuality in the speech and language problems children can experience [27] which this study and most previous ones fail to take into account. Future research should attempt to explore the influences on particular speech or language difficulties in early childhood, albeit that this will necessitate large samples. These might be better conducted through longitudinal analysis of administrative datasets in health and education of children referred for speech, language and communication problems [3,14,28].

Although the prevalence rates reported for Ireland may be small when applied to the child population, sizeable numbers of children within primary or elementary schools will have persistent speech and language difficulties. Previous research has shown the impact on children’s literacy and numeracy [4]. However, teachers may feel ill-equipped to address these difficulties within the classroom or to advise parents accordingly [21].

Referrals to speech and language therapists is the main alternative in more affluent countries to supporting children with speech, language and communication needs and their families. In Ireland, our data suggests that around 50% of the children with speech and language difficulties had seen a therapist within the last 12 months and the percentage may have been higher over a longer time period. Lower figures were reported for Australia [4]. At age 5, half of children who no longer had difficulties at age 9, were reported to have had a single consultation; presumably for assessment, whereas a higher proportion of children with continuing difficulties at age 9 had two or more contacts when the child was aged 5. At nine years of age, all but 10% of the improved group reported no further contacts in the previous 12 months, whereas those with difficulties at age 9 had a similar level of contact as at age 5 with one quarter of all the children having two or more contacts with a speech and language therapist. This data suggests that current provision of speech and language therapy within Ireland is insufficient to meet the needs of children and to provide guidance to primary caregivers and teachers as to how they can assist the children’s speech and language development [29,30]. Moreover, the impact of early intervention is well established [31] and the potential of parent-implemented interventions is promising, with training and support for parents and appropriate intensity of intervention [20].

## 5. Conclusions

The prevalence of speech and language difficulties reported by the primary caregivers of Irish children decreases from one in 6 at age 5 to one in 12 at age 9. However, one in 20 children were reported to have difficulties at both ages. Compared to children who no longer had speech and language difficulties at age 9, these children were more likely to have two or more developmental impairments as well as current or past hearing impairments. Teachers and parents also reported a greater number of social-emotional difficulties for children with persistent speech and language difficulties. Family characteristics did not differ significantly across the two groupings. However, only a relatively small amount of variance was explained by the model which suggests high individual variation among children in the difficulties experienced and their personal circumstances. At best, up to only one third of the children at ages 5 and 9 with speech and language difficulties had two or more contacts with a speech and language therapist in the preceding 12 months. Increased support to these children, their parents and their teachers would seem to be warranted.

## Figures and Tables

**Table 1:** The number and percentage of children with a speech and language difficulty at ages 5 and 9 and the percentage decrease for each type of difficulty.

Type of Speech and Language Difficulty	Age 5	Age 9	Percentage Decrease
Lisps or difficulty pronouncing certain letter combinations	825 (10.7%)	331 (4.3%)	61%
Speech not clear to others	742 (9.7%)	268 (3.5%)	64%
Speech is developing slowly	410 (5.3%)	190 (2.5%)	54%
Speech not clear to family	390 (5.1%)	161 (2.1%)	61%
Difficulty finding words	350 (4.6%)	256 (3.3%)	28%
Difficulty putting words together	331 (4.3%)	225 (2.9%)	35%
Voice sounds unusual	218 (2.8%)	106 (1.4%)	54%
Stutters, stammers	164 (2.1%)	100 (1.3%)	42%
Reluctant to speak	118 (1.5%)	69 (0.9%)	44%
Other (including limited or no speech)	67 (0.9%)	54 (0.7%)	24%
Totals	1281	623	51%

**Table 2:** The number and percentage of children by the number of speech and language difficulties reported when the children were 5 and 9 years old.

Age 5	Age 9			Total
	No Difficulties	1–2 Difficulties	3+ Difficulties	
No difficulties	6176 (80.3%)	150 (2.0%)	83 (1.1%)	6409 (83.4%)
1–2 difficulties	558 (7.3%)	92 (1.2%)	39 (0.5%)	689 (9.0%)
3+ Difficulties	337 (4.4%)	95 (1.2%)	158 (2.1%)	590 (7.7%)
Total	7071 (92.0%)	337 (4.4%)	280 (3.6%)	7688 (100.0%)

**Table 3:** The contrasts between children who had improved at age 9 compared to those with difficulties at age 9.

Variable		Improved (*n* = 882)	Difficulties (*n* = 588)	Statistical Tests
Developmental problems	None One Two +	77.1% 16.2% 6.7%	60.4% 22.0% 17.6%	Chi Sq 60.34, df 2, *p* < 0.001 Cramer’s V 0.200 ^+^
Hearing Problem	Currently In past None	2.3% 3.2% 94.4%	6.2% 7.6% 86.2%	Chi Sq 30.41, df 2, *p* < 0.001 Cramer’s V 0.142 ^+^
Socio-emotional problems Primary carer rating: Cut-off14	Below Above	81.4% 18.6%	63.8% 36.2%	Chi Sq 59.29 df 1, *p* < 0.001 Cramer’s V 0.198 ^+^
Socio-emotional problems Teacher’s rating: Cut-off14:	Below Above	85.6% 14.4%	70.2% 29.8%	Chi Sq 48.03, df 1, *p* < 0.001 Cramer’s V 0.188 ^+^
Socio-emotional problems Primary Carer’s rating: Cut-off 17	Below Above	90.3% 9.7%	74.5% 25.5%	Chi Sq 66.82 df 1, *p* < 0.001 Cramer’s V 0.210 ^+^
Socio-emotional problems Teacher’s rating: Cut-off 17:	Below Above	91.2% 8.8%	82.4% 17.6%	Chi Sq 21.37 df 1, *p* < 0.001 Cramer’s V 0.131 ^+^
Number of friends child has	0 or 1 2+	8.6%	17.1%	Chi Sq 24.63 df 1, *p* < 0.001
		91.4%	82.9%	Cramer’s V 0.128^+^
Primary carer reports child bullied	Yes No	23.1%	31.9%	Chi Sq 14.58 df 1, *p* < 0.001
		76.9%	68.1%	Cramer’s V 0.098 ^+^
Primary carer’s rating—Conflict		15.68 (SD 6.51)	17.82 (SD 7.20)	t(1235.40) = −5.91, *p* < 0.001 Cohen’s D 0.315 ^^^
Teacher’s rating—Conflict		12.39 (SD 5.61)	13.80 (SD 6.47)	t(1013.71) = −4.05, *p* < 0.001 Cohen’s D 0.236 ^+^
Primary Carer’s Stress score		13.49 (SD 4.60)	14.88 (SD 4.73)	t(1484) = −5.66, *p* < 0.001 Cohen’s D 0.299 ^^^
Primary Carer’s Depression Score		2.58 (SD 3.31)	3.07 (SD 3.66)	t(1220.35) = −2.67, *p* < 0.01 Cohen’s D 0.143 ^+^

^+^ Weak effect size: ^ Moderate effect size.

**Table 4:** The results of the step-wise Binary Logistic Regression contrasting children with improved speech and language difficulties and those with continuing difficulties.

Variables	B	S.E.	Sig.	Exp(B)	95% C.I. for EXP(B) Lower Upper	
**BLOCK 1: Chi Sq 126.24 df 7 *p* < 0.001: Nagelkerke R Square = 0.130**						
Current or past hearing problem	0.892	0.222	0.004	2.439	1.579	3.768
One developmental Impairment	0.578	0.151	0.000	1.782	1.324	2.397
Two + developmental impairments	1.155	0.210	0.000	3.173	2.103	4.786
Higher social-emotional problems –Primary carer—Age 9	0.490	0.172	0.004	1.632	1.164	2.289
Level of conflict with Primary Caregiver—Age 9	0.005	0.011	0.654	1.005	0.984	1.027
Parental stress scores for Primary Caregiver—Age 9	0.042	0.015	0.006	1.043	1.012	1.074
Total depression score for Primary Caregiver—Age 9	−0.027	0.059	0.648	0.973	0.866	1.093
Constant	−1.471	0.221	0.000	0.230		
**BLOCK 1 + 2: Chi Sq 132.99 df 9 *p* < 0.001: Nagelkerke R Square = 0.137**						
Current or past hearing problem	0.872	0.224	0.000	2.392	1.543	3.709
One developmental Impairment	0.554	0.153	0.000	1.739	1.290	2.346
Two + developmental impairments	1.134	0.213	0.000	3.109	2.048	4.721
Higher social-emotional problems –Primary carer—Age 9	0.409	0.176	0.020	1.505	1.067	2.124
Level of conflict with Primary Caregiver—Age 9	0.005	0.011	0.646	1.005	0.984	1.027
Parental stress scores for Primary Caregiver—Age 9	0.040	0.015	0.009	1.040	1.010	1.072
Total depression score for Primary Caregiver—Age 9	−0.037	0.060	0.533	0.963	0.857	1.083
Child has 2 or more close friends	0.166	0.210	0.427	1.181	0.783	1.781
Primary Carer report that child was bullied in past year	0.347	0.140	0.014	1.414	1.074	1.862
Constant	−1.512	0.223	0.000	0.220		
**BLOCK 1 + 2 + 3: Chi Sq 148.797 df 11 *p* < 0.001: Nagelkerke R Square = 0.152**						
Current or past hearing problem	0.859	0.225	0.000	2.361	1.520	3.668
One developmental Impairment	0.468	0.158	0.003	1.597	1.171	2.177
Two + developmental impairments	1.059	0.219	0.000	2.884	1.878	4.430
Higher social-emotional problems –Primary carer—Age 9	0.339	0.183	0.063	1.404	0.982	2.008
Level of conflict with Primary Caregiver—Age 9	0.007	0.011	0.535	1.007	0.985	1.029
Parental stress scores for Primary Caregiver—Age 9	0.041	0.015	0.008	1.041	1.010	1.073
Total depression score for Primary Caregiver—Age 9	−0.030	0.060	0.621	0.971	0.862	1.092
Child has 2 or more close friends	0.096	0.214	0.654	1.101	0.724	1.674
Primary Carer report that child was bullied in past year	0.335	0.142	0.018	1.397	1.059	1.845
Higher social-emotional problems –Teacher—Age 9	0.796	0.202	0.000	2.216	1.491	3.294
Level of conflict with Teacher—Age 9	−0.255	0.111	0.022	0.775	0.623	0.964
Constant	−0.770	0.402	0.055	0.463		

**Table 5:** The percentage of children who had contact in past 12 months with a speech and language therapist (SLT) at age 5 and age 9 for those who had improved and those experiencing difficulties at age 9.

Contact with SLT		Improved (*n* = 895)	Difficulties (*n* = 619)	Statistical Tests
Contacts at age 5	None One contact Two + contacts	450 (50.3%) 229 (25.6%) 216 (24.1%)	316 (51.1%) 100 (16.2%) 203 (32.8%)	Chi Sq 24.94 df 2 *p* < 0.001 Cramer’s V 0.128 ^+^
Contact at age 9	None One contact Two + contacts	813 (90.8%) 35 (3.9%) 47 (5.3%)	347 (56.0%) 113 (18.3%) 159 (25.7%)	Chi Sq 247.76 df 2 *p* < 0.001 Cramer’s V 0.405^^^

## Data Availability

The data used in this study is available in request from the Irish Social Sciences Database: https://www.ucd.ie/issda/.
